# Exploring the adaptive mechanism of *Passiflora edulis* in karst areas via an integrative analysis of nutrient elements and transcriptional profiles

**DOI:** 10.1186/s12870-019-1797-8

**Published:** 2019-05-06

**Authors:** Mengxuan Xu, Anding Li, Yao Teng, Zimou Sun, Meng Xu

**Affiliations:** 1 Co-Innovation Center for Sustainable Forestry in Southern China, Nanjing Forestry University, Nanjing, 210037 China; 2 College of Forestry, Nanjing Forestry University, Nanjing, 210037 China; 3 Institute of Mountain Resources, Guizhou Academy of Sciences, Guiyang, 550001 China

**Keywords:** *Passiflora edulis*, rocky desertification, Passion fruit, Nutrient element, Transcriptome, Adaptability

## Abstract

**Background:**

*Passiflora edulis*, known as passion fruit and native to South America, is now widely cultivated throughout southern China for its edible value, medicinal efficacy and ornamental properties. We have developed a cold-tolerant variety of *P. edulis* (‘Pingtang 1’) that can survive subzero temperatures and is highly adaptable in Karst areas. In this study, cuttings of ‘Pingtang 1’ were cultivated in a limestone (L) rocky desertification area and a sandy dolomite (D) rock desertification area. Changes in nutrient elements in both the soils and plants were revealed in the two plots. Moreover, RNA sequencing (RNA-Seq) was performed to profile the root transcriptomes for further exploration of nutrient adaptative mechanism of *Passiflora edulis* in Karst regions.

**Results:**

In this study, a total of, 244,705,162 clean reads were generated from four cDNA libraries and assembled into 84,198 unigenes, of which 56,962 were annotated by publicly available databases. Transcriptome profiles were generated, and 1314 unigenes (531 upregulated and 801 downregulated) were significantly differentially expressed between the L and D root cDNA libraries (L_R and D_R, respectively); these profiles provide a global overview of the gene expression patterns associated with *P. edulis* adaptability to Karst soils. Most unigenes including a number of differentially expressed genes (DEGs) were involved in nutrient element uptake, utilization, signal regulation. And DEGs enriched in KEGG pathways of plant hormone signal transduction, phenylpropanoid biosynthesis, and biosynthesis of unsaturated fatty acids were significantly expressed.

**Conclusion:**

These results could contribute to better understanding the adaptation of this species to environmental stress and thus enhance the potential for successfully introducing and commercially deploying *P. edulis*.

**Electronic supplementary material:**

The online version of this article (10.1186/s12870-019-1797-8) contains supplementary material, which is available to authorized users.

## Background

*Passiflora*, a genus within the Passifloraceae family, comprises approximately 500 species, most of which are distributed throughout Latin America; many species of this genus are widely cultivated for their edible value, medicinal efficacy and ornamental properties [1–3]. In the early twentieth century, *Passiflora edulis* Sims (also known as passion fruit), the newest entry on the fruit market at the time, was introduced in southern China, mainly in Taiwan, Guangdong, Guangxi and Fujian. This exotic and perennial vine is adapted to warm and humid climates and is thus generally intolerant of cold temperatures. However, we have developed a cold-tolerant variety of *P. edulis* (‘Pingtang 1’) that can survive subzero temperatures and is well adapted to the Karst habitats of Guizhou.

Karst topography is characterized as a typical landscape shaped from the dissolution of soluble rocks, such as limestone (L), dolomite (D), and gypsum [4]. As one of the most fragile terrestrial ecosystems [5, 6], Karst landforms are especially developed and widespread in soluble rock areas, such as the Nullarbor Plain in Australia, the Western Highland Rim in the United States, and the Yunnan-Guizhou Plateau in China. In Southwest China, Karst rocky desertification has become a serious threat to local agriculture, forestry, and livestock husbandry [7, 8], and Karst soils have poor water-holding capacity and nutrient cycling ability. The new *P. edulis* variety ‘Pingtang 1’ is widely cultivated in the Karst areas of Guizhou. Because of breeding and popularization, this variety has undergone several rounds of testing under extreme low temperature conditions and is well adapted to Karst soils despite their poor water-holding capacity and soil nutrient cycling ability [9, 10]. However, information regarding the regulatory patterns and signaling pathways of genes in this exotic and perennial vining species related to adaptation to Karst soils remains unknown.

Because of the rapid development of next-generation sequencing (NGS) technology and the completion of whole-genome sequencing projects for many plant species [11, 12], increased availability of genomic and transcriptomic data has provided insights into root-soil interactions. *P. edulis*, also known as passion fruit and native to South America, is now widely cultivated throughout southern China for its edible fruit, medicinal efficacy and ornamental properties. In this study, cuttings of ‘Pingtang 1’ were cultivated in L rocky desertification areas and sandy D rocky desertification areas. Nutrient elements of both plants (roots, stems and leaves) and soils in the two plots were systematically analyzed. Furthermore, transcriptome libraries from the roots were constructed and sequenced, and 1314 DEGs (531 upregulated and 801 downregulated) were identified as being significantly differentially expressed between the L and D root cDNA libraries (L_R and D_R, respectively). Most differentially expressed genes (DEGs) were involved in nutrient element uptake and utilization and signal regulation. DEGs in KEGG pathway of plant hormone signal transduction, phenylpropanoid biosynthesis, and biosynthesis of unsaturated fatty acids may reveal the potential responses of plant growth and chilling-resistance in two soil sites. The results provide a global overview of gene expression patterns associated with *P. edulis* adaptability to Karst soils and could contribute to revealing the adaptive mechanism at the molecular level.

## Results

### Nutrient elements in soils and plants

Analyses of nutrient elements were based on the average values of three biological replications. Yellow soil is characterized as being acidic; the soils of the L and D plots had pH values of 6.45 and 5.75, respectively, both of which are lower than 7.0, indicating the acidic properties of the two soils.

**Fig. 1:**
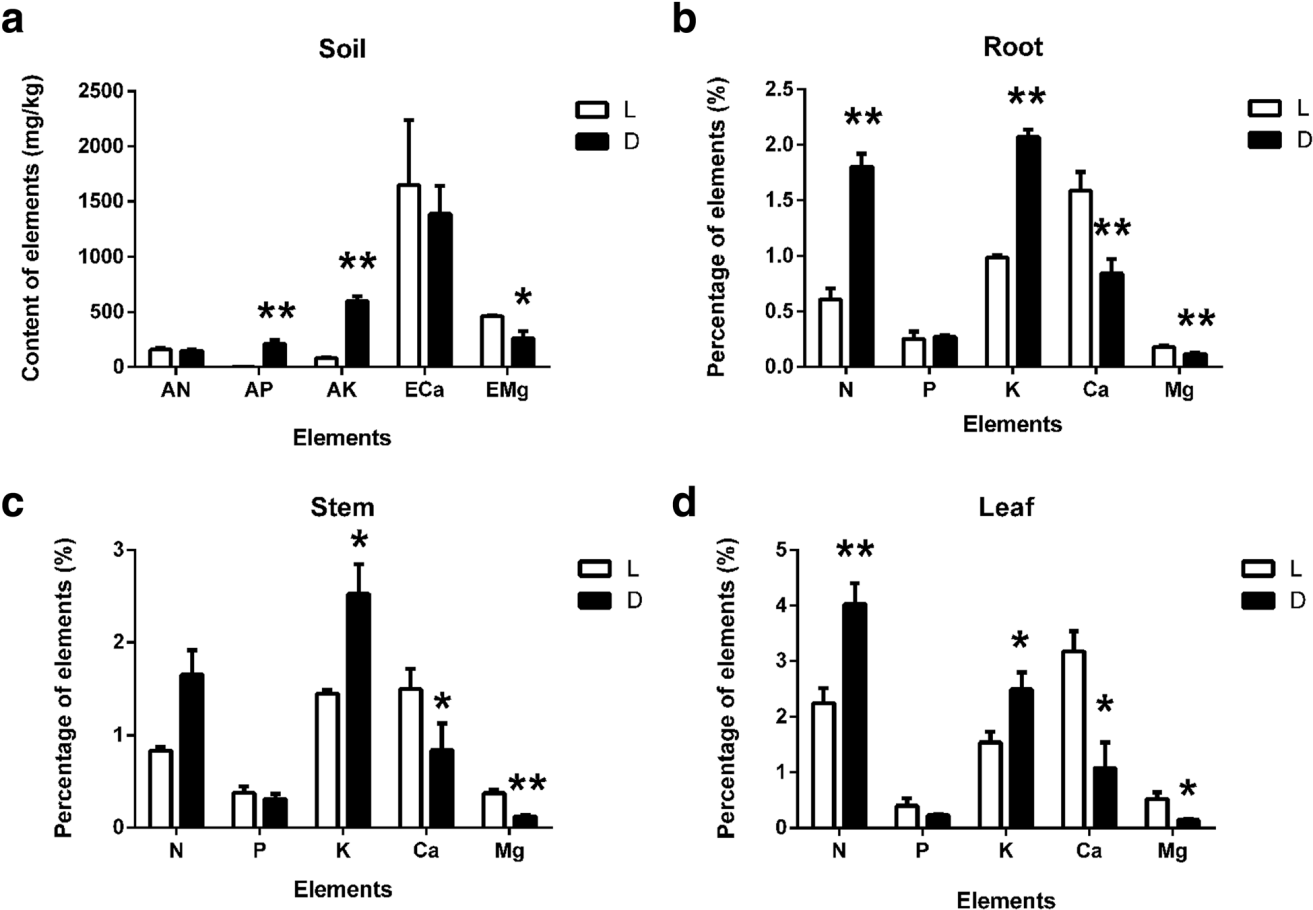
**a**) The nutrient element (AN: alkali hydrolyzable nitrogen, AP: available phosphorus, AK: available potassium, ECa: exchangeable calcium, EMg: exchangeable magnesium) concentrations in the soils at two sites (L and D) (mg/kg); **b**) Percentage of nutrient element (N: nitrogen, P: phosphorus, K: potassium, Ca: calcium, Mg: magnesium) concentrations in the roots at the two sites (L and D); **c**) Percentage of nutrient element (N, P, K, Ca and Mg) concentrations in the stems; **d**) Percentage of nutrient element (N, P, K, Ca and Mg) concentrations in the leaves. One asterisk in the figure indicates a significant difference in elements in the roots or other tissues between sites D and L (*p* < 0.05), while two asterisks indicate a highly significant difference (*p* < 0.01)

For soil nutrient elements, the amount of available nitrogen (AN) in two sites were similar, exchangeable calcium (ECa), and exchangeable magnesium (EMg) at the L site were greater than those at the D site. However, the available potassium (AK) content exhibited the opposite trend (Fig. 1a), and the available phosphorus (AP) content was much higher at the D site than at the L site (Fig. 1a). The difference in EMg between the two soil samples was significant (*p* < 0.05), and the differences in AP and, AK were highly significant (*p* < 0.01).

Generally, the proportions of elements in plant tissues indicated that the total amount of N, K, and Ca were greater than P and Mg (Fig. 1b, c, d). The amounts of N and K were higher in the D site plants than in the L site plants, while the amounts of Ca, and Mg were higher in the L site plants (Fig. 1b, c, d). The differences in N, K, Ca, and Mg in the roots growing in the two soil types were highly significant (*p* < 0.01) (Fig. 1b). The stems from the two soil types presented highly significant (*p* < 0.01) Mg concentrations, while the K and Ca concentrations were significantly different (*p* < 0.05) (Fig. 1c). In the leaves, N was highly significant (*p* < 0.01), while K, Ca, and Mg were significant (*p* < 0.05) (Fig. 1d).

### RNA sequencing and transcriptome assembly

A total of 253,078,350 raw reads and 244,705,162 clean data were obtained from the four cDNA libraries after sequencing. The mean Q30 and GC bias values were 90.31 and 44.92%, respectively, indicating high-quality sequencing (Table 1). After sequence assembly, 142,677 transcripts ranging from 201 to 16,965 bp in length were obtained. The N50 of all transcripts was 2046 bp, while that of 84,198 unigenes was 2326 bp. The proportion of transcripts whose length ranged from 200 to 500 bp accounted for more than half of all the transcripts, and the different length intervals of the unigenes were evenly distributed (Table 2).

**Table 1:** Summary of RNA-seq data from four RNA libraries of two-year-old root of ‘Pingtang 1’ from two different soil sites in Karst habits

Libraries	Raw Reads	Clean Reads	Clean Bases	Q30 (%)	GC (%)
L_R1	63,256,218	61,695,416	9.25G	92.99	44.74
L_R2	60,417,094	58,150,918	8.72G	87.53	45.18
D_R1	66,971,474	65,129,330	9.77G	92.80	45.20
D_R2	62,433,564	59,729,498	8.96G	87.93	44.57
Summary	253,078,350	244,705,162	36.7G

**Table 2:** Distribution of length interval of transcripts and unigenes obtained from the RNA-seq of roots of ‘Pingtang 1’

Length distribution	Transcripts		Unigenes	
	Number	Percentage	Number	Percentage
200–500	80,937	56.73%	23,428	27.82%
500-1 k	22,545	15.80%	21,588	25.64%
1-2 k	18,547	13.00%	18,534	22.01%
> 2 k	20,648	14.47%	20,648	24.52%
Total	142,677		84,198	
Total nucleotide	135,742,661		119,695,604	
Mean length	951		1422	
N50	2046		2326	

### Gene annotation and functional classification

**Fig. 2:**
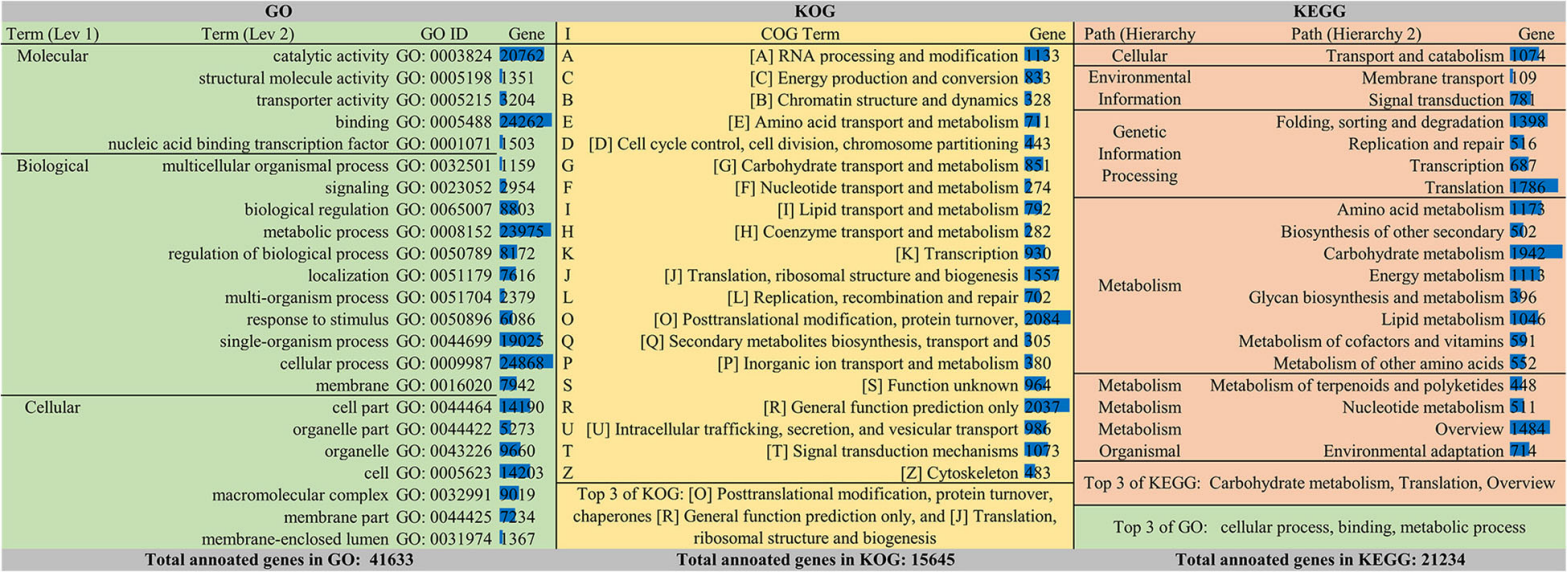
Unigenes enriched in the hierarchical levels of GO, KOG and KEGG terms. The top 20 terms of each classification are included with their corresponding numbers of unigenes

There were 52,229, 41,877, 41,644, 41,034, 40,640, 21,234, and 15,645 significantly matched unigenes in the NR, SwissProt, GO, NT, Pfam, KO, and KOG databases, respectively, with successful annotation percentages of 62.03, 49.73, 49.45, 48.73, 48.26, 25.21, and 18.58%, respectively. The NR database yielded the greatest proportion of matched unigenes, while the KOG database yielded the lowest. A total of 9093 unigene hits were universally obtained across the seven databases tested (Table 3). Based on the annotations, the unigenes were further classified into different groups, with 41,633, 15,645, and 21,234 unigenes enriching 50, 25, and 19 terms for the GO, KOG, and KEGG databases, respectively (Fig. 2). GO analysis revealed enrichment in cellular processes, metabolic processes (biological process [BP]), binding, catalytic activity (molecular function [MF]), and cell and cell parts (cellular component [CC]), thus underlying three additional significant terms (single-organism process (BP), organelle (CC) and macromolecular complex). For the KOG analysis, the top three terms were [O] posttranslational modification, protein turnover, and chaperones; [R] general function prediction only; and [J] translation, ribosomal structure and biogenesis. Considering the potential relationship of gene expression in the different soil types, we focused on the fourth term ([T] signal transduction mechanisms) for further analysis. The KEGG analysis provided a feasible reference for linking transcriptomes to biological systems by pathway mapping. We focused on the following top five terms: carbohydrate metabolism; translation; overview of metabolism; folding, sorting and degradation; and amino acid metabolism.

**Table 3:** Unigenes annotated to the seven databases

Component	Number of Genes	Percentage (%)
Annotated in NR	52,229	62.03
Annotated in NT	41,034	48.73
Annotated in KO	21,234	25.21
Annotated in SwissProt	41,877	49.73
Annotated in PFAM	40,640	48.26
Annotated in GO	41,644	49.45
Annotated in KOG	15,645	18.58
Annotated in all Databases	9093	10.79
Annotated in at least one Database	56,962	67.65
Total Unigenes	84,198	100

### Identification and functional analysis of DEGs

**Fig. 3:**
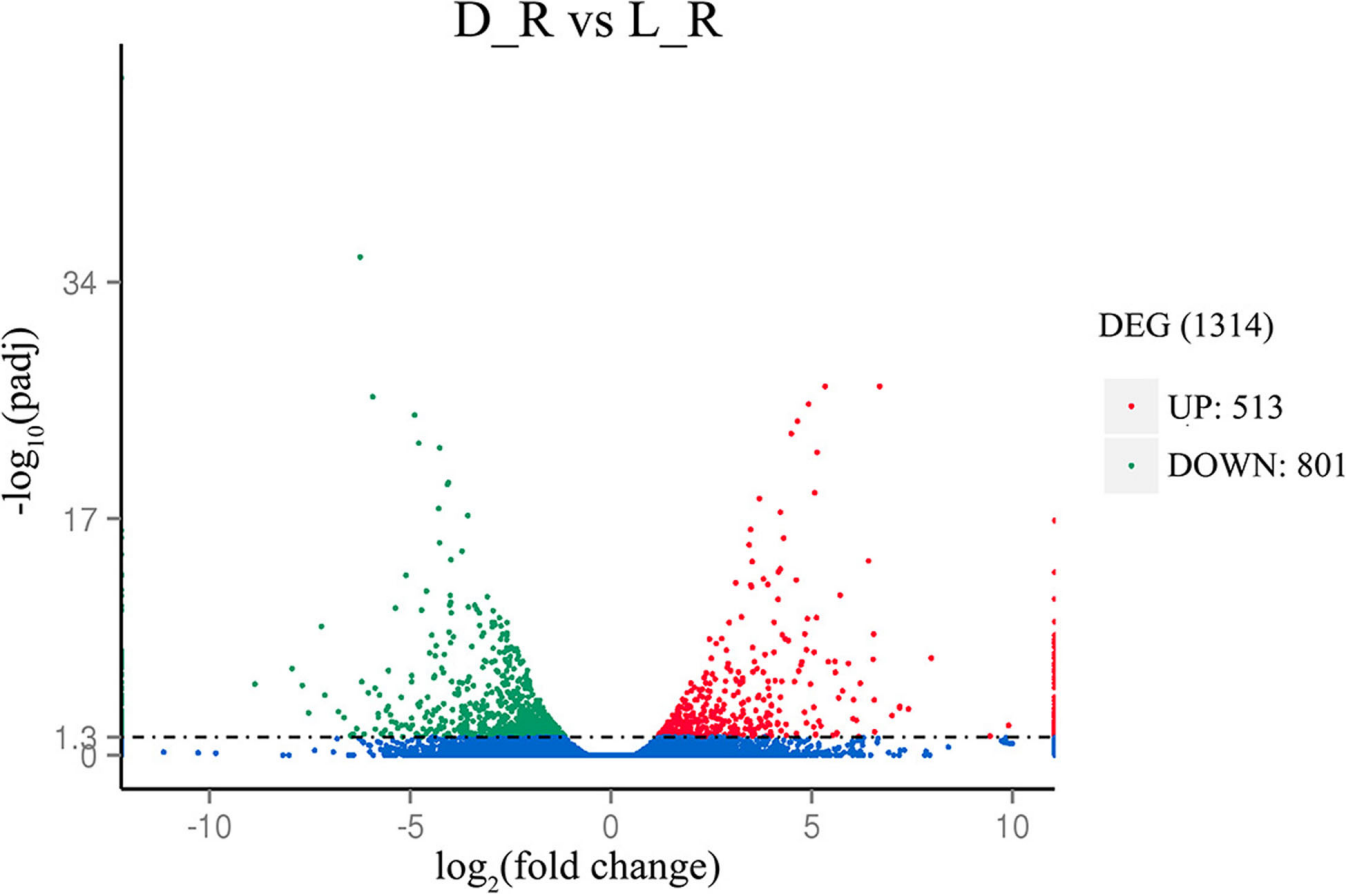
Analysis of significant DEGs between D_R and L_R. The red and green dots indicate the expression of unigenes that are up- and downregulated in the D_R sample. The volcano plot shows the log_2_(fold change) value, while -log_10_(padj) values are shown on the ordinate. Smaller corrected *p*-values (padj) are correlated with greater corresponding -log_10_(padj) values. Therefore, the points in the upper-left and upper-right sections represent the down- and upregulated genes whose expression levels differed significantly, respectively. With the criterion of padj< 0.05 for filtering, the red and green dots in the figure are greater than 1.3, and 1314 DEGs were identified. Among them, 513 and 801 DEGs were up- and downregulated, respectively, between D_R and L_R

The read counts of each unigene were sorted by RSEM and transformed to the expected number of fragments per kilobase of transcript sequence per millions base pairs sequenced (FPKM) to characterize the expression levels of the genes. On average, the FPKM value of 73.64% of the unigenes in four libraries was greater than 0.3, which was the minimum value of the subsequent gene expression level analysis. Moreover, the FPKM intervals were 15–60 and > 60, which signified that the medium and high levels of gene expression were 8.11 and 2.71%, respectively. Using the criterion of padj< 0.05 for filtering, 1314 DEGs were identified, among which 513 and 801 DEGs were up- and downregulated, respectively (D_R vs L_R, respectively; Fig. 3). All the DEGs were generated by comparing the expression of unigenes in soil sites D to L (D Vs. L). The up/down regulated DEGs referred to the higher/lower expression of unigenes in plant of soil site D.

**Fig. 4:**
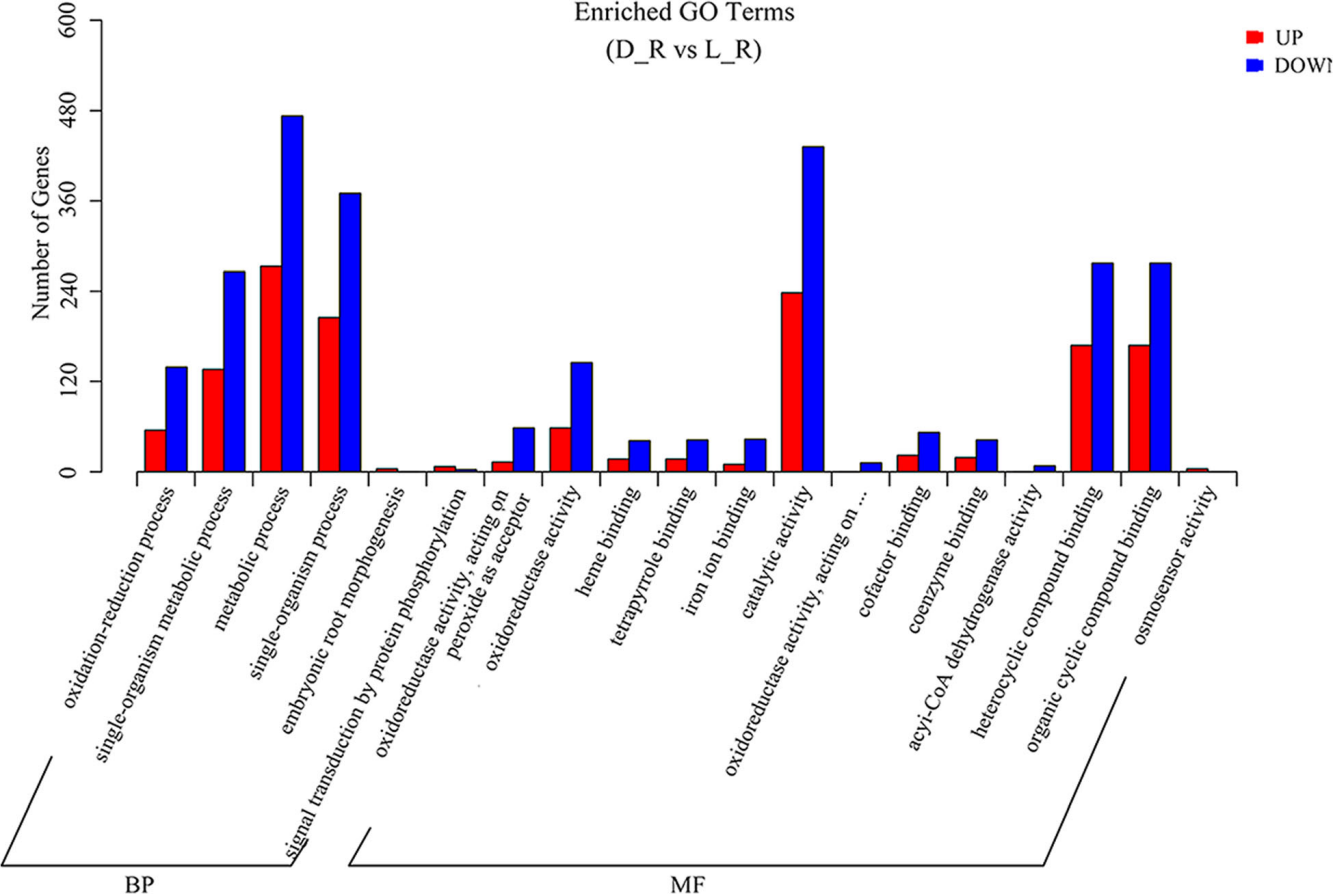
Illustration of the passion fruit root DEG enrichment of 19 terms with BP and MF categories. In the figure, “oxidoreductase activity, acting on” indicates “oxidoreductase activity, acting on paired donors, with oxidation of a pair of donors resulting in the reduction of molecular oxygen to two molecules of water”

The DEGs clearly enriched in each GO term are shown in Fig. 4. DEGs were enriched in 19 BP and MF terms, and the top three enriched terms were metabolic process (BP), catalytic activity (MF), and single-organism process (BP); these terms were associated with 746, 670 and 575 DEGs, respectively, which were equal to percentages of 69.46, 62.38, and 53.54%, respectively. Directed acyclic graphs (DAGs) can intuitively reveal the GO terms of enriched DEGs and their hierarchical relationship. We performed enrichment analysis for the up- and down-regulated DEGs based on their BP and MF, respectively.

**Fig. 5:**
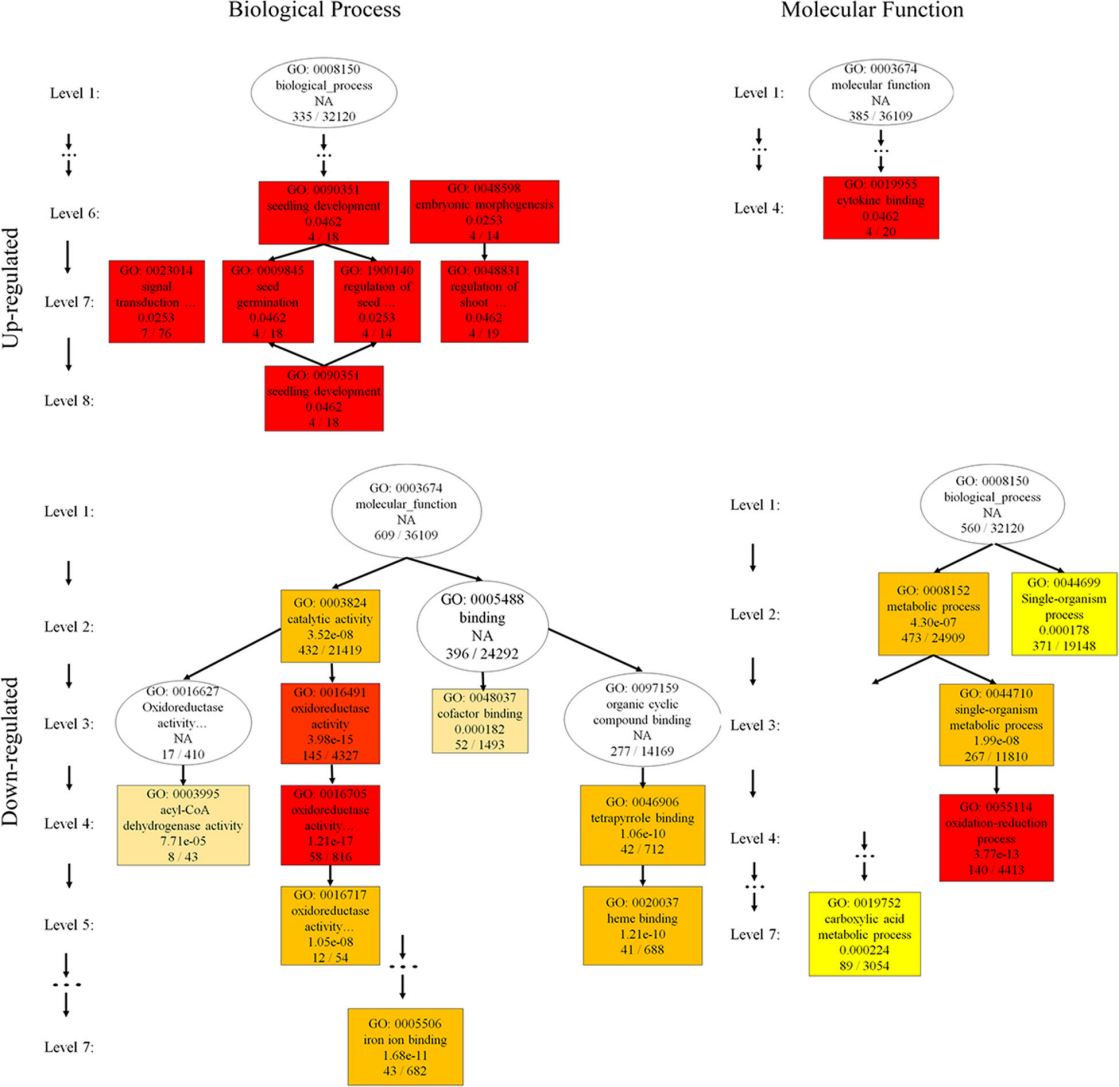
DAG showing the results of DEG enrichment according to GO analysis. The top ten enrichments were selected in each category of two GO classifications. The main node is described in the boxes, and deeper colors reveal a higher degree of enrichment. Terms with low intermediate expression are not shown. The DAGs were divided into four categories: upregulation in BP (**a**) and MF (**b**) and downregulation in BP (**a**) and MF (**b**)

The DAGs of the upregulated DEGs (Fig. 5) revealed 4 and 8 levels for MF and BP, respectively. Cytokine binding (GO: 0019955) in level 4 was the only term classified as MF. For BP, six terms were enriched in four sublevels. Signal transduction by protein phosphorylation (GO: 0023014) and regulation of shoot system development (GO: 0048831) at level 8 and embryonic morphogenesis (GO: 0048598) at level 6 were enriched separately without enriched parent nodes. Seed germination (GO: 0010029) was a sub-node of both seed germination (GO: 0009845) and regulation of seedling development (GO: 1900140) at level 7; these two terms were subnodes of seedling development (GO: 0090351) at level 6.

The downregulated DEGs of 10 terms were enriched in each DAG, which were layered with 7 and 8 levels of MF and BP, respectively. For MF, except level 6, the DEGs were enriched in terms at every level. Three branches were significantly enriched, representing oxidoreductase activity, with the parent node of catalytic activity (GO: 0003824); the second was iron ion binding (GO: 0005506), and the third was tetrapyrrole binding (GO: 0046906) to heme binding (GO: 0020037). With respect to the BP ontology, the DEGs were significantly enriched in the oxidation-reduction process (GO: 0055114); their parent node was associated with the single-organism metabolic process (GO: 0044710). There were also some DEGs enriched in terms involved in cellular amino acid metabolic processes.

**Fig. 6:**
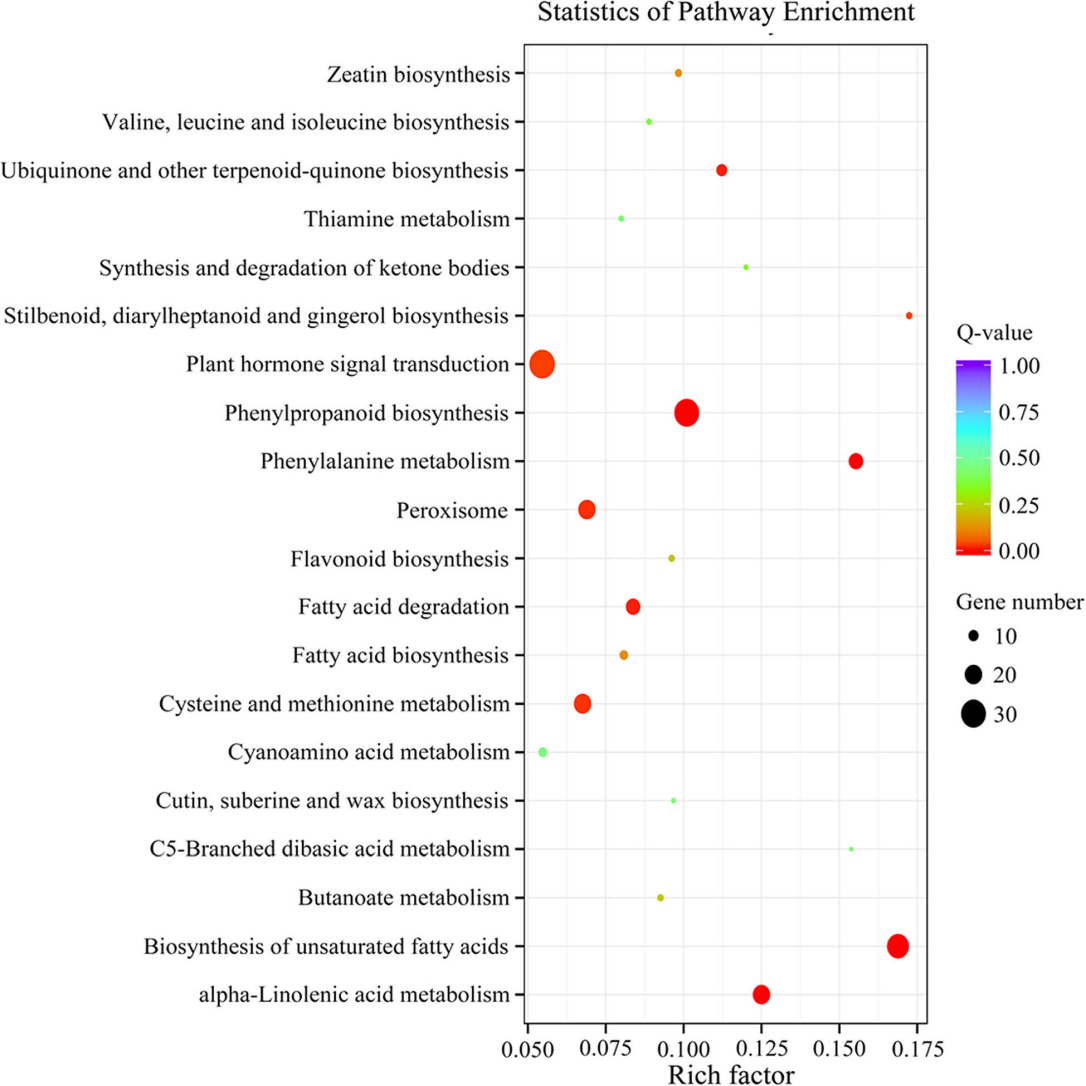
The degree of enrichment was measured by the rich factor, Q-value and numbers of enriched genes in the pathways. The top 20 pathways with the highest degree of enrichment are shown. The rich factor is the ratio between the number of enriched DEGs and the number of all annotated genes in the pathway. The Q-value indicates the p-value after multiple hypothesis test correction and ranges from 0 to 1. A higher enrichment is achieved when the Q-value approaches 0

Following analysis of the clearly enriched DEGs, the KEGG classification results were presented as a scatter plot (Fig. 6). For significantly enriched DEGs, the top three pathways were plant hormone signal transduction (31 DEGs), phenylpropanoid biosynthesis (30 DEGs), and biosynthesis of unsaturated fatty acids (26 DEGs), with Q-values of 0.025, 2.78e-06 and 2.81e-09, respectively. The pathways of alpha-linolenic acid metabolism (20 DEGs), phenylalanine metabolism (16 DEGs), ubiquinone and other terpenoid-quinone biosynthesis (11 DEGs), fatty acid degradation (16 DEGs), peroxisomes (20 DEGs), and cysteine and methionine metabolism (20 DEGs) were significantly enriched. The specific information of DEGs enriched in KEGG pathways were showed in supplement file (Additional file 5: Table S1).

**Fig. 7:**
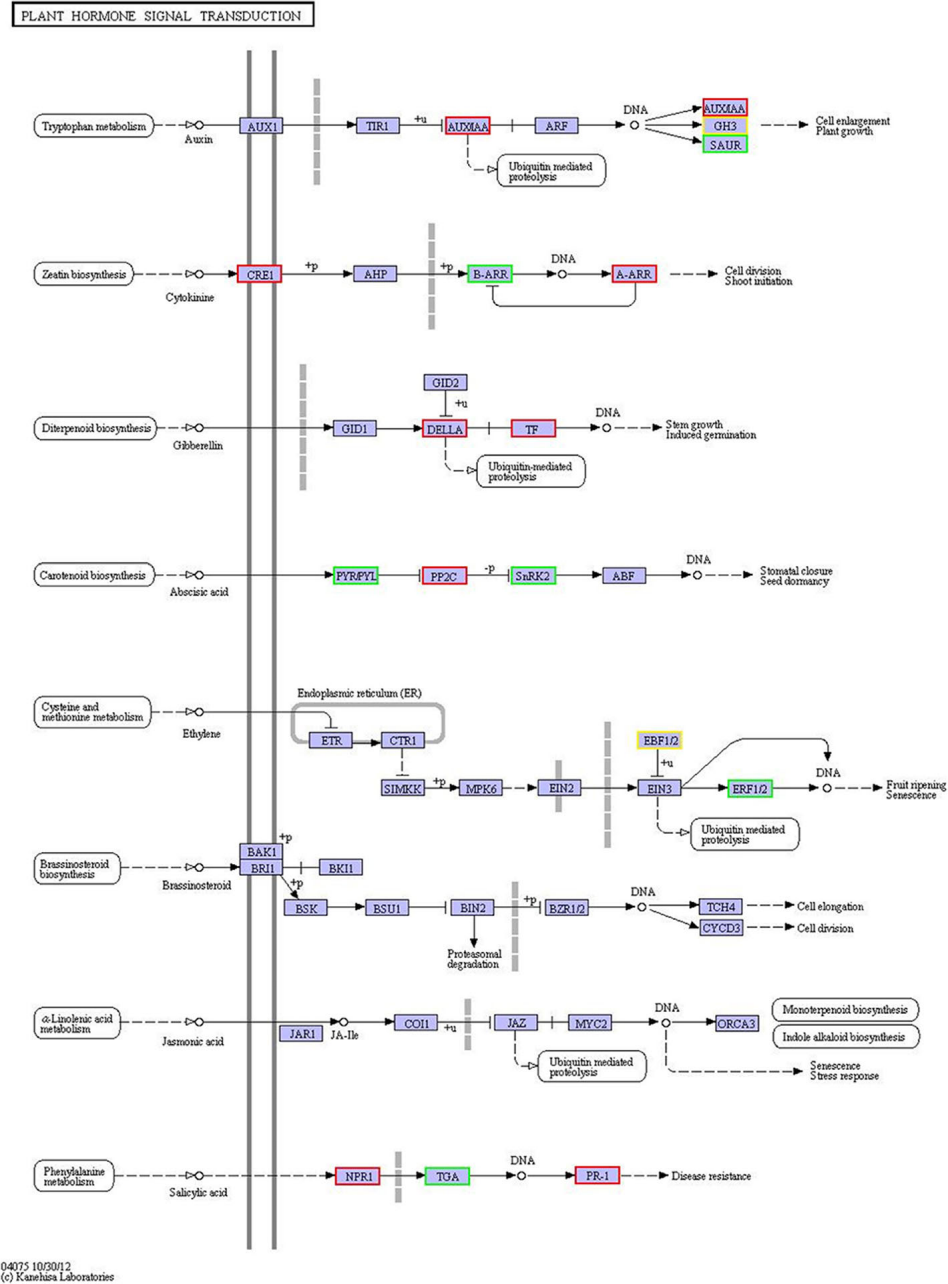
KEGG pathway enrichment analysis of plant hormonal signal transduction pathways. DEGs were enriched in 6 of the 8 subpathways. The red highlighting indicates that a DEG was upregulated, the green indicates that a DEG was downregulated, and yellow indicates both

The KEGG enrichment analysis revealed that nine pathways were significantly enriched, with the top three representing plant hormone signal transduction, phenylpropanoid biosynthesis and biosynthesis of unsaturated fatty acids. Thirty-one DEGs were enriched in six sub-pathways of plant hormone signal transduction (with 568 background unigenes): 6 DEGs were enriched in auxin signal transduction, 8 in cytokinin signal transduction, 3 in gibberellin signal transduction, 5 in abscisic acid (ABA) signal transduction, 3 in ethylene (ETH) signal transduction, and 6 in salicylic acid (SA) signal transduction (Fig. 7). Thirty of the DEGs enriched involved in phenylpropanoid biosynthesis pathway (Additional file 1: Figure S1) and 26 involved in the biosynthesis of unsaturated fatty acids pathway were downregulated (Additional file 2: Figure S2).

### qPCR validation of DEGs

**Fig. 8:**
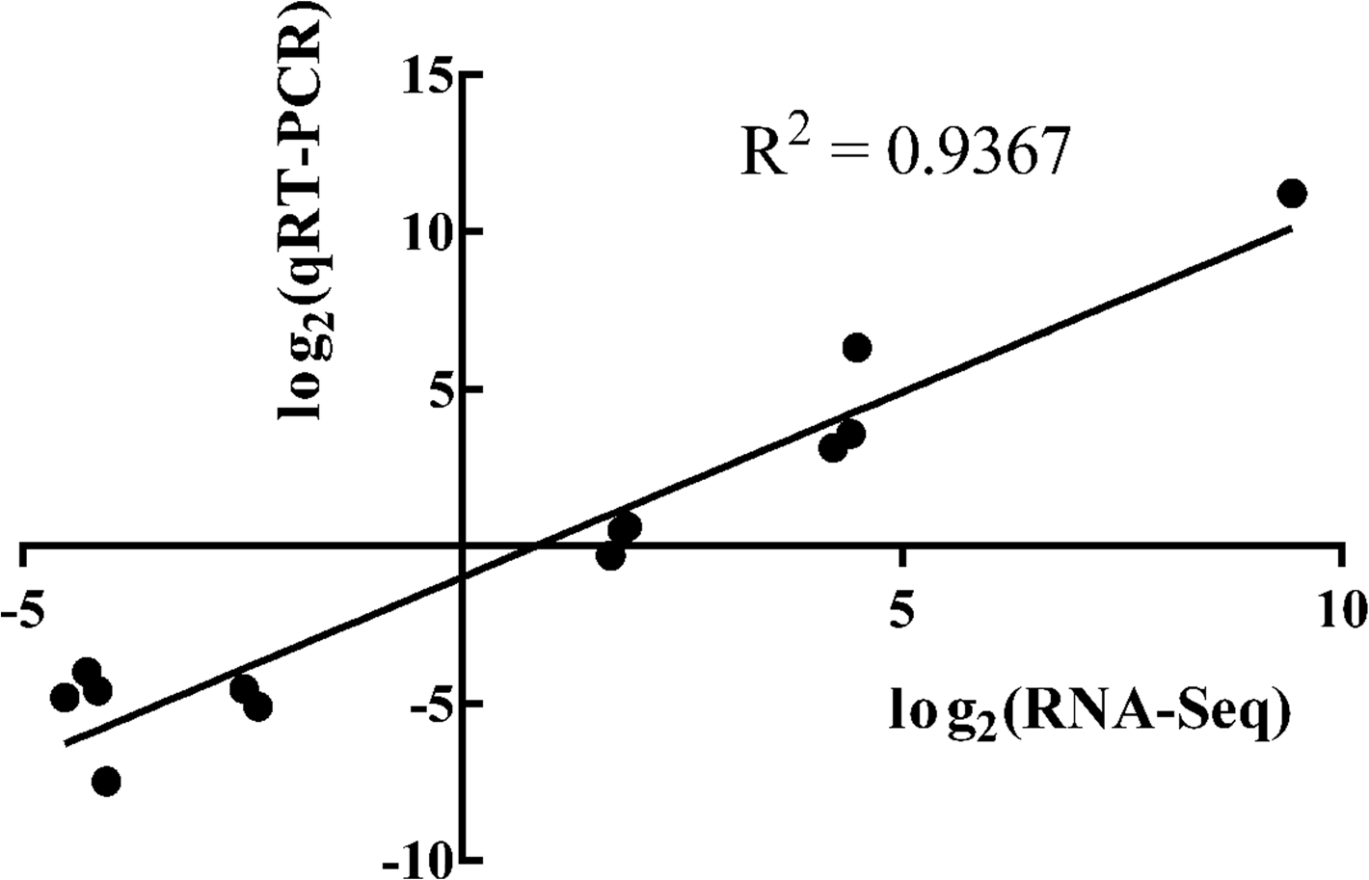
Scatter plot based on the log_2_(fold changes) of the RNA-Seq and qPCR data. The abscissa represents the log_2_(fold change) value of the read counts from RNA-Seq, while the ordinate indicates the log_2_(fold changes) of the negative delta-delta Ct values from qPCR. The value of R^2^ = 0.9367 (*p* < 0.01) indicates the significant similarity of the results between RNA-Seq and qPCR, which demonstrated the accuracy of RNA-Seq

We randomly selected 13 DEGs for qPCR assessment to validate the accuracy of the Illumina RNA sequencing (RNA-Seq) results, and DEGs mentioned in discussion were all verified. These DEGs presented an abundance of increased or decreased transcripts between root samples from the two different soils. For qPCR, the level of gene expression was determined by the relative quantity (RQ). Compared to RNA-Seq, the RQ results were consistent with the expression patterns of target genes assessed via qPCR. In addition, we used fold changes to quantify the relevance of the RNA-Seq expression levels and qPCR results. Figure 8 shows a scatter plot of the RNA-Seq data and the qPCR results as log_2_(fold changes), indicating significant similarity (R^2^ = 0.9367, *p* < 0.01) between the results of the two methods and verifying the accuracy of the RNA-Seq. On the other hand, the trend of RQ results of DEGs listed in discussion in two soil sites was mostly consistent with the prediction of RNA-Seq (Additional file 3: Figure S3 and Additional file 4: Figure S4).

## Discussion

### DEGs involved in nutrient uptake, transport, and transduction

Roots play a very important role in plant anchorage, water and nutrient uptake, and the synthesis and storage of metabolites [13]. N, P, and K are essential nutrient elements and play an indispensable role in plant growth and development. In plants, P is an important component of metabolites and macromolecules, such as ATP, phospholipids and nucleic acids, and is involved in many biological pathways, such as those related to gene expression and signal transduction [14]. Orthophosphoric acid (H_2_PO_4_^−^, HPO_4_^2−^ and PO_4_^3−^) is the main form of absorption in plants, and there is a severe shortage of P in agricultural soils worldwide. The P starvation response aims to achieve the self-balance of P via a series of physiological and biochemical activities, including the absorption, transportation and reuse of P. Under low P conditions, plant roots activate insoluble P in the soil by secreting organic acids (OAs) to release Pi from refractory P compounds in the soil and increase the content of AP [15]. Previous studies have shown that the activity of phosphoenolpyruvate carboxylase (PEPC) was increased under conditions of P deficiency, providing a carbon source for the synthesis of OAs [16, 17]. The results of those studies showed that 27 unigenes were homologous with the PEPC family protein, and one DEG of phosphoenolpyruvate carboxylase kinase 2 (PPCK2) was significantly upregulated. The activity of PEPC is regulated mainly by phosphoenolpyruvate carboxylase kinases (PPCKs) [18]. The low phosphatase conditions in the soil of the L plots probably stimulated the metabolic activities in the passion fruit plants and promoted the synthesis of OAs for the transformation of insoluble P in the soil.

Adaptation to low P environments and P procurement from the environment require a complex signaling network in plants [19, 20], which is dependent on the P transport system. P transporters (PHT) play an important role in the absorption and transportation of P [21] and are grouped into five families: PHT1, PHT2, PHT3, PHO1 and PHO2. In this study, 137 unigenes, associated to 28 PHT1, 3 PHT2, and 25 PHO1, were homologous to those of P transporter families. Among those unigenes, the expression of PHO84, a DEG encoding the protein of a high-affinity P transporter [22], was significantly higher in the roots from the L site soil than in the roots from the D site soil (Table 4). The results from the present study showed that the amount of AP in the L site soil (0.0026 g/kg) was considerably lower than that in the D site soil (0.2115 g/kg). However, the P content in plants grown at the two sites was approximately equal. The expression of P transporters was inconsistent with the trend of element changes in both the plants and soils. The upregulation of P transporter genes improved the absorption of P by roots at the site L soil.

**Table 4:** The characteristics and expression of DEGs associated with nutrient elements uptake, utilization, and signal regulation

Gene_id	Annotation	FPKM(D_R)	FPKM(L_R)	D_R vs L_R
Cluster-7898.36820	*PHO84*	21.895	57.785	Down
Cluster-7898.4433	*APS1*	3.89	15.175	Down
Cluster-7898.45233	*PAP1*	4.685	13.32	Down
Cluster-7898.18934	*PAP1*	0.225	3.12	Down
Cluster-7898.27691	*PPCK2*	12.32	39.205	Down
Cluster-7898.61262	*POT5*	6.98	33.955	Down
Cluster-7898.5070	*AKT1*	2.775	9.135	Down
Cluster-7898.5365	*HAK5*	0.755	6.74	Down
Cluster-7898.26747	*CLC-b*	11.79	2.735	Up
Cluster-7898.53478	*CLC-b*	3.535	0.355	Up
Cluster-7898.46597	*NIPA*	14.01	48.875	Down
Cluster-7898.49803	*CML11*	78.065	414.37	Down
Cluster-7898.61829	*CBL7*	2.355	19.595	Down
Cluster-7898.46341	*GLR2.7*	0.54	3.12	Down
Cluster-7898.5855	*GRXC6*	2.76	27.335	Down
Cluster-7898.61518	*CIPK14*	1.59	10.925	Down
Cluster-7898.2597	*CIPK8*	4.4425	5.4625	Down

The functional information of DEGs listed were showed in supplemental files (Additional file 6: Table S2)

In addition, Pi starvation induces the synthesis of acid phosphatases (APases) in plants. In roots, APases are important for the hydrolysis and mobilization of Pi from extracellular phosphomonoesters for plant nutrition [23]. In the present study, 116 unigenes were annotated to APase genes, and one *ASP* and two *PAP1* genes were significantly upregulated in the roots from the site L soil (Table 4). A low P environment reportedly induces the transcription of the *PAPs* [24]. The results of the present study showed that *P. edulis* could increase the activity of APases to improve the efficiency of P utilization and the ability of P absorption to adapt to a low P stress environment. These responses may play an important role in the stability of P balance in plants.

Plants contain a large amount of K, which exists in an ionic form within cell sap or is adsorbed on the surface of protoplast colloids. K^+^ plays an important role in maintaining the balance of cell potential and osmotic potential, and K is absorbed through K channels and K^+^ transporter in plants [25]. In this study, 219 unigenes were annotated to K transport-related proteins, including 111 K channel genes and 79 K^+^ transporter genes; among the K^+^ transporter unigenes, 15 were annotated to high-affinity K^+^ transporters (HAKs). The amount of K in the root samples at site D was clearly greater than that at site L. However, DEG analysis revealed that the expression of the *HAK5*, *POT5*, and *AKT1* genes was downregulated. The expression model was not consistent with the changes in K in the plants and soils at sites D and L (Table 4). Previous studies showed that *HAK5* [26] and *AKT1* [27] were inducible in a high-affinity K^+^ uptake system under low K conditions, which may suggest that the low concentration of K in the site L soil mediates the expression of these genes for K uptake.

Alkali hydrolyzable N consisted of inorganic N (ammonium [NH_4_^+^]-N, nitrate [NO_3_^−^]-N) and hydrolytic organic N (amino acids, amides and hydrolyzable proteins). NH_4_^+^-N and NO_3_^−^-N are the two major N sources for higher plants, and NO_3_^−^ is the most abundant source of N [28]. NH_4_^+^ AMT transporters are the major pathways for the uptake of NH_4_^+^ in roots [29]. In the present study, 29 unigenes were homologous to NH_4_^+^ transporters, including 16 AMT type genes. However, the expression of these unigenes was not markedly different between the roots at the two soil sites. On the other hand, four gene family members were shown to be involved in NO_3_^−^ uptake, allocation, and storage in higher plants: NO_3_^−^ transporter 1/peptide transporter (NRT1/PTR), NRT2, chloride channel (CLC), and slow anion channel-associated 1 homolog 3 (SLAC1/SLAH) [30]. With respect to the unigene annotations, 75 unigenes were homologous to NO_3_^−^ transport-related genes, including 61 *NRT1*/*PTR*s, 4 *NRT2*s, and *5 CLC*s. Differential expression analysis revealed that 2 *CLC-b* genes were expressed at higher levels in roots from site D than in roots from site L (Table 4). In addition, the results showed a tendency toward similarity in the amount of AN in the soil; however, the concentration differed between plants, with a clear increase in plant growth at site D compared with that at site L. The uptake of NO_3_^−^ in plants is influenced by many factors; NO_3_^−^-N is absorbed faster in lower-pH soil solution, and nutrient elements, such as P [31] and K [32], promote the absorption of N. The results possibly indicated that the relatively low pH and relatively high concentrations of P, K, and Ca may have increased the expression of NO_3_^−^ transport-related genes in the roots at site D, influencing the intracellular exchange of NO_3_^−^.

Mg^2+^ plays an important role in the physiological processes of plant growth and development. For plant adaptation to different soil conditions, members of two Mg transporter families have been reported to regulate intra- and extracellular Mg^2+^ transport in higher plants: CorA-like Mg^2+^ transport systems [33] and Mg/proton exchangers [34]. In the present study, 93 unigenes were annotated to Mg^2+^ transport, among which 26 CorA-like Mg^2+^ transporters and 5 Mg/proton exchangers were identified. One DEG encoding the Mg^2+^ transporter *NIPA* was downregulated in the roots of the soil at site D compared with that at site L (Table 4). Moreover, the concentration of Mg^2+^ in the plants and soils was higher at site L than at site D, and the expression pattern was in accordance with the trend of the Mg^2+^ content in the roots and soils, which may indicate that the gene expression level tended to be positively regulated by the changes in elements.

As a universal signaling messenger, Ca^2+^ is an essential nutrient element of plants and regulates many physiological processes in plant cells [35]. Hyperpolarization-activated Ca^2+^ channels (HACCs), depolarization-activated Ca^2+^ channels (DACCs,), Ca^2+^-permeable outward-rectifying K^+^ channels (KORCs), and voltage-insensitive cation channels (VICCs) are four important Ca^2+^ channels in the plasma membrane [36]. In the present study, 134 unigenes were annotated to Ca^2+^ channel genes, including 30 annexin, 2 *TPC1*, 6 *SKOR*, 1 *GORK*, 41 *CNGC*, and 52 *GLR* genes. Of these genes, those coding for Ca^2+^ channels were highly expressed under high concentrations of Ca ions in the soils at both sites. Specifically, 59 unigenes were filtered after they were standardized to FPKM> 3; among them, 6 unigenes with an FPKM value > 60 were significantly expressed, and 27 unigenes were filtered by 15 < FPKM< 60. The highly expressed unigenes related to Ca^2+^ channels suggested that the concentration of Ca^2+^ in the cytoplasm was increased under high Ca concentrations.

The soils of Karst areas are based on dissolved Ca-rich parent rock, and the native plants in those regions have evolved a special Ca ion adaptation system [4]. Therefore, this system could be the source of the adaptation mode for ‘Pingtang 1’ plants growing on Karst landforms. Roots take up Ca via Ca^2+^ channels and transport it through Ca^2+^-ATPase and Ca^2+^/H^+^ antiporters to maintain low concentrations of Ca in the cytoplasm [30]. Plants that have adapted to Ca-rich environments by enriching the amount of Ca ions can transport those ions and store them via Ca^2+^/H^+^ reverse transporters and Ca^2+^-ATPases. Our results showed that 113 unigenes were Ca^2+^-ATPase genes; among these genes, 33 were filtered according to the FPKM> 3 standard, while 8 were highly expressed, with FPKM values> 15. Moreover, 26 unigenes were homologous to Ca^2+^/H^+^ antiporters, and 9 were highly expressed, with FPKM values > 3. These findings suggest that the high expression levels of Ca^2+^-ATPase and Ca^2+^/H^+^ antiporters may be responsible for maintaining low Ca concentrations in the cytoplasm.

In response to varied extracellular Ca^2+^ levels, relatively constant cellular concentrations of Ca^2+^ suggested a regulatory mechanism for Ca^2+^ homeostasis [37]. Ca^2+^-sensing receptors (CASs), which are localized at the plasma membrane, may be primary transducers of extracellular Ca^2+^ in plants [38]. In this study, 7 unigenes were homologous to CASs, and 3 of these genes, whose FPKM values were > 3, were expressed. Ca-dependent protein kinase (CDPK), calmodulin (CaM) and CaM B-like protein (CBL) are three major Ca^2+^ receptors involved in signal transduction. CDPKs comprise four types of subfamilies: CDPKs, CDPK-related proteins (CRKs), CaM-dependent protein kinases (CaMKs), and Ca^2+^ and CaM-dependent protein kinases (CCaMKs). CaM transduces Ca^2+^ signals through CaM-binding protein (CaMBP) [39], and CBL transduces Ca^2+^ signals only by interacting with CBL-interacting proteins (CIPKs) [40]. Our results revealed 106 predicted CaMs, 32 CaMKs, 67 CDPKs, 29 CDMKs, 16 CRKs, 11 CCaMKs, 24 CBLs and 51 CaMBPs in the libraries. Of these genes, 67 were filtered according to the FPKM> 3 standard. The expression patterns of the Ca^2+^ receptor genes revealed that high Ca^2+^ concentrations in the cytoplasm mediated downstream reactions in cells. On the other hand, the expression of DEGs annotated to Ca^2+^ channels (*GLR2.7*, *GRXC6*), Ca^2+^ signal transport proteins and downstream receptors (*CIPK14*, *CIPK8*, *CML11*, *CBL7*) was downregulated in the roots of the soil at site D compared with that at site L (Table 4). Considering the consistency of Ca^2+^ changes in plants and soils, Ca^2+^ absorption and utilization in cells may tend to be positively regulated by elemental changes in the soil environment.

In this study, at the transcriptional level, differential analysis of element transporter genes revealed that the uptake, transport and utilization of nutrient elements were affected by the elemental conditions at the soil sites. The changes in the amounts of elements in the plants were not consistent with the changes in the soils at the two sites, which may indicate that interactions occurred between elements when the plants responded to the different soil environments. Furthermore, the expression patterns of DEGs involved in the plant hormone signal transduction pathway (Fid. 7) suggested that the responses of plants were different in the soils of different nutrient element contents. The expression pattern of genes related to Ca^2+^ transport and transduction may suggest that Ca-rich conditions induce a series of responses concerning Ca uptake, transport, and downstream reactions of Ca^2+^ receptors, which occur to maintain a dynamic Ca balance within ‘Pingtang 1’ plant cells to allow adaption to Karst areas. These results will contribute to revealing the adaptive mechanism.

### DEGs enriched in top-three significant KEGG pathways

In the study, ‘Pingtang 1’ was a specifically cold-resistant variety of *P. edulis* in karst areas. It was necessary to research the mechanism of adaptability to low temperature in two soil sites of karst areas. From the results of DEGs in KEGG enrichment, we focused the sight on the top-three significant pathways, which were plant hormone signal transduction, phenylpropanoid biosynthesis, and biosynthesis of unsaturated fatty acids. On this basis, combining with the mineral elements condition of soil and plant in two soil sites, there were some analysis and suggestions.

In previous researches, cold-resistances of plants were associated with the components of the fatty acid of the plasma membrane, the stability of cell structure would be improved by increasing membrane lipid unsaturation [41]. DEGs in the pathway of biosynthesis of unsaturated fatty acids were all down-regulated. Several DEGs were homologous with FAD2, which encodes the enzyme that is essential for polyunsaturated lipid synthesis in the endoplasmic reticulum [42]. Hence, the differential expression of these DEGs related to lipid unsaturation may influence the responses to low temperature of plants.

As for phenylpropanoid biosynthesis pathway, DEGs involved in the biosynthesis of cinnamaldehyde, p-coumaryl alcohol, caffeylalcohol, 5-Hydroxyconiferyl alcohol, and sinapyl alcohol were obviously down-regulated, however, DEGs encoded peroxidase [EC:1.11.1.7] were both up-regulated and down-regulated. Those alcohols were involved in the biosynthesis of secondary metabolism, the secondary metabolic process was believed to be the result of adaptation in dealing with the relationship between plants and the ecological environment. And therefore, the down-regulated DEGs may decrease the synthesis of related secondary products and further affect potential adaptability of *P. edulis* in karst areas. Research has shown exogenous K elements would diminish the expression of gene 4CL and F5H [43]. And the amount of AK of soil and tissues in site D were lower than D, which may indicate the association with significantly down-regulated expression of 4CL and F5H.

In the pathway of plant hormone signal transduction, auxin regulates plant growth and development by altering the expression of diverse genes [44], while DEGs related to three auxin-responsive AUX/IAA and SAUR families were both up-regulated, and SAUR and GH3 were down-regulated in the results. In cytokinin signaling, the type-B response regulators mediate cytokinin signal transduction (B-ARR) [45] were down-regulated. And the other type response regulator (A-ARR) was up-regulated, which was reported that could negatively regulate cold resistance of plants [46]. In the research, DEGs of ARR-A would possibly induce the further responses of chilling-resistance of ‘Pingtang 1’ in soil site L. In the downstream pathway of Gibberellin (GA), DELLA, as a receptor of GA [47], the higher expression of which in soil site D may suggest the positive impact to regulatory effects of GA in site D’s plant. Abscisic acid inhibits type 2C protein phosphatases (PP2C) via the PYR/PYL family, and high PP2C activity may prevent phosphorylation and activation of SnRK2s and downstream factors [48]. And moreover, SnRK2s could phosphorylate the ABA-responsive element binding factor family to activate ABA-responsive genes [49], which would further encode protective proteins and enzymes for osmolyte synthesis for stress signal transduction in plants [50]. DEGs related to PYL and SnRK2 were down-regulated, while PP2C were up-regulated in abscisic acid sub-pathway. The biosynthesis of ethylene was induced by abiotic stress, such as chemicals, metals, and extreme temperatures [51, 52]. In signal pathway leading from salicylic acid (SA), NPR1 was a regulatory linkage between TGA factors and PR genes [53]. Up-regulated DEGs annotated to NPR1 and RP-1 proteins were and down-regulated TGA may suggest us the differential responses to stress for plant defense in two kinds of soil sites. Comprehensively, pathways of plant hormone signal transduction, phenylpropanoid biosynthesis, and biosynthesis of unsaturated fatty acids participate in complicated physiological processes of plants. The expression pattern of these DEGs may influence the downstream responses about plant growth, development, adaptivity to the environment, and the resistance to stress.

‘Pingtang 1’ were reported both adaptive to the cold condition and different soil types in karst areas in southwestern of China [10], in our study, the physiological characteristics of two cutting plants grew in two soil sites were similar after carefully observation. Many factors existed in microenvironments would influence the plant growth and development, and the effect from nutrient elements on the adaptation of *P. edulis* to environmental stress was partly limited. In view of the impact of the environmental condition such as terrain, light, annual precipitation, and human activity, the two adjacent soil sites with different soil types were selected for minimizing the interference of other factors while designing the experiment. On this basis, combined with the connection of different elements concentration of soil and plant in two site plots, the key point was concentrated on the analysis of molecular mechanism about the adaptability of passion fruits in karst areas. However, the influence of other environmental factors was inevitable, this research was our first step to study the adaptive mechanism of passion fruits in karst areas, and we would like to consider more factors in the next researches for further exploration.

## Conclusion

‘Pingtang 1’, a new variety of *P. edulis*, which is highly adaptable in Karst areas. After integrated analysis upon nutrient elements differences in both the soils and plants and transcriptomic profile in plant roots, the results of DEGs expression patterns related to utilization, transport and transduction of mineral elements, and further analysis about DEGs in top-three enriched pathways will support for understanding to exploring the adaptive mechanism of plants.

## Methods

### Plant materials and growth conditions

‘Pingtang 1’, the cold-tolerant variety of *P. edulis*, was massively propagated by cuttings and served as the research material. Cuttings of ‘Pingtang 1’ were cultivated in L rocky desertification areas and sandy D rocky desertification areas. The two plots are located in Kedu town, Pingtang Country, Guizhou Province (25°43′0.53″N, 106°48′19.48″E, altitude 852 m above sea level, mid-subtropical humid monsoon climate). Soil samples were collected from three randomly selected locations in each test plot. The roots, stems and leaves of 2-year-old plants were sampled as three biological replicates per test plot. Two root samples selected from those three biological replicates for RNA extraction were immediately frozen in liquid nitrogen (N) and stored at − 80 °C in an ultralow temperature freezer. We declare plant materials (*P. edulis*) and the field culture used in the research complied with government regulations.

### Determination of nutrient elements

Soil nutrient elements were detected as described in the most recent Agriculture Industry Standard of China (2015). Briefly, the soil pH was measured in a water:soil solution (1:2.5) via a pH meter. AP was extracted in accordance with the molybdenum antimony colorimetry method at a soil solution ratio of 1:10, and AN was determined using the alkaline hydrolysis diffusion method after reduction with FeSO_4_ and Zn. With respect to the medicinal preparation of ammonium acetate, AK was extracted by the flame photometry method, while ECa and EMg were extracted by the atomic absorption spectrophotometry method.

The proportions of the N, K, P, Ca, and Mg elements in the roots, stems, and leaves were determined in 2-year-old plants growing in the two plots. After H_2_SO_4_-H_2_O_2_ digestion, the N content was determined by the semimicro alkali distillation method, while the molybdate yellow colorimetric method and the flame spectrophotometric method were used to determine the contents of P and K, respectively. The atomic absorption spectrophotometry method was used to determine the total Ca, and Mg was determined after digestion by dry ashing. The whole-plant proportion of each element was calculated by the weighted average of three sets of samples.

### RNA isolation, cDNA library construction and sequencing

The total RNA of the roots was isolated using a RNeasy Plant Mini Kit (Qiagen, Hilden, Germany). The RNA quality was monitored via 1% agarose gel electrophoresis and via an Agilent Bioanalyzer 2100 (Agilent Technologies, CA, USA). The cDNA libraries of the L plot were named L_R, and those of the D plot were named D_R.

The four cDNA libraries were generated using an NEBNext Ultra RNA Library Prep Kit (NEB, USA) according to the manufacturer’s instructions (Illumina). According to the manufacturer’s recommendations, clustering of the index-coded samples was performed on a cBot Cluster Generation System using TruSeq PE Cluster Kit v3-cBot-HS (Illumina, USA). The prepared libraries were sequenced, and 150-bp paired-end reads were generated (Illumina HiSeq 2500, USA). The raw images were transformed using CASAVA base-calling in the FASTQ format (raw data), and all raw reads were deposited into the National Center for Biotechnology Information (NCBI) Sequence Read Archive (SRA accession no. SRP150688).

### Transcriptome assembly and annotation

Clean data (clean reads) were generated by filtering adapter sequences and low-quality reads that consisted of more than 10% poly-N or contained more than 50% of bases that had a Q-value ≤20. All downstream analyses were based on high-quality clean data. Transcriptome assembly was achieved using Trinity version r20140413p1 [54] based on left.fq and right.fq, with the min_kmer_cov value set to 2 by default and all other parameters set to their default. The clean reads of various isoforms of one gene were assembled into distinct transcripts, but the same subcomponent, which could be considered a gene, and the longest transcript of each subcomponent were defined as “unigenes” for annotation. All assembled unigenes were queried via BLAST against the Nr, Nt, Pfam, Clusters of Orthologous Groups of proteins (KOG/COG), SwissProt, Kyoto Encyclopedia of Genes and Genomes (KEGG) Orthology (KO) and Gene Ontology (GO) databases using the Blast2GO program with a cutoff E-value of 10^− 6^.

### Differential expression analysis

The relative expression levels of the unigenes were estimated by mapping clean reads to the Trinity transcriptome assembly using RNASeq by Expectation Maximization (RSEM) [55] with the trimmed mean of M-values method. Differential expression analysis of the unigenes was performed using read counts with the DESeq R package. The resulting *p*-values were adjusted to Q-values to compensate for multiple hypothesis testing. Unigenes with an adjusted Q-value < 0.05 (reported by DESeq) were assigned as DEGs. A volcano plot of the DEGs was constructed using the log_10_(padj) and log_2_(fold change) values. GO and KEGG enrichment analyses of the DEGs were implemented using the GOseq R package and KOBAS software [39], respectively.

### Quantitative real-time polymerase chain reaction (qPCR)

Total RNA was isolated from various samples and treated with RNAse-free DNase I. The DNase-treated RNA was reverse transcribed using random hexamer primers; the specific primers were designed to generate amplification fragments of 70–150 bp. Quantitative real-time polymerase chain reaction (qPCR) was performed on an ABI ViiA 7 Real-Time PCR platform using FastStart Universal SYBR Green Master with ROX in accordance with the manufacturer’s protocol; all reactions were performed in triplicate. The relative expression levels were calculated using the delta-delta Ct method, and *HIS* was used as a housekeeping gene [10].

## Additional files

Additional file 1: **Figure S1.** Down-regulated DEGs enriched in phenylpropanoid biosynthesis pathway. (JPG 343 kb)

Additional file 2: **Figure S2.** Down-regulated DEGs involved in the biosynthesis of unsaturated fatty acids pathway. (JPG 220 kb)

Additional file 3: **Figure S3.** The validation of DEGs listed in Table 4. The figure combined bar graph of gene expression and the scatter diagram of correlation analysis between RNA-seq and qPCR. DEGs involved were mentioned in Table 4. (JPG 469 kb)

Additional file 4: **Figure S4.** The validation of DEGs in KEGG pathways. The figure combined bar graph of gene expression and the scatter diagram of correlation analysis between RNA-seq and qPCR. DEGs involved were enriched in the pathways of biosynthesis of unsaturated fatty acids, phenylpropanoid biosynthesis and plant hormone signal transduction, respectively. (JPG 392 kb)

Additional file 5: **Table S1.** The annotation of DEGs listed in Table 4. (XLS 55 kb)

Additional file 6: **Table S2.** The details of DEGs enriched in KEGG pathways. (XLS 28 kb)

## Abbreviations

4CL: 4-coumarate-CoA ligase
AK: Available potassium
AN: Available nitrogen
AP: Available phosphorus
CAD: Cinnamyl-alcohol dehydrogenase
DAGs: Directed acyclic graphs
DEGs: Differentially expressed genes
ECa: Exchangeable calcium
EMg: Exchangeable magnesium
F5H: Ferulate-5-hydroxylase
FPKM: Fragments per kilobase of transcript sequence per millions base pairs sequenced
NGS: Next-generation sequencing
OAs: Organic acids
