# Supplementary figures and images for: Exploring the adaptive mechanism of Passiflora edulis in karst areas via an integrative analysis of nutrient elements and transcriptional profiles
Source: BMC Plant Biol. 2019 May 6;19:185. doi: 10.1186/s12870-019-1797-8 (PMC6503377; doi:10.1186/s12870-019-1797-8)

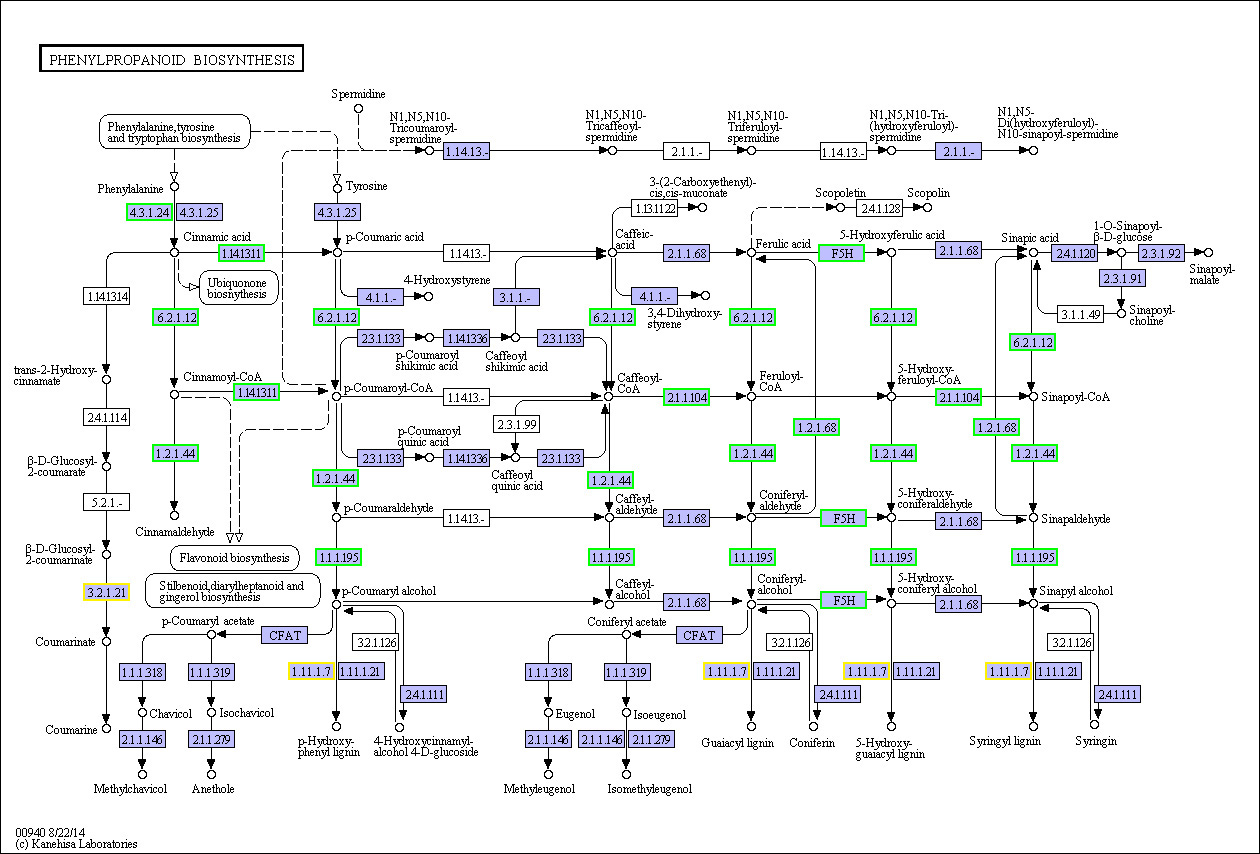

Supplement: Supplementary file 1 — Figure S1. Down-regulated DEGs enriched in phenylpropanoid biosynthesis pathway. (JPG 343 kb) [file 12870_2019_1797_MOESM1_ESM.jpg]

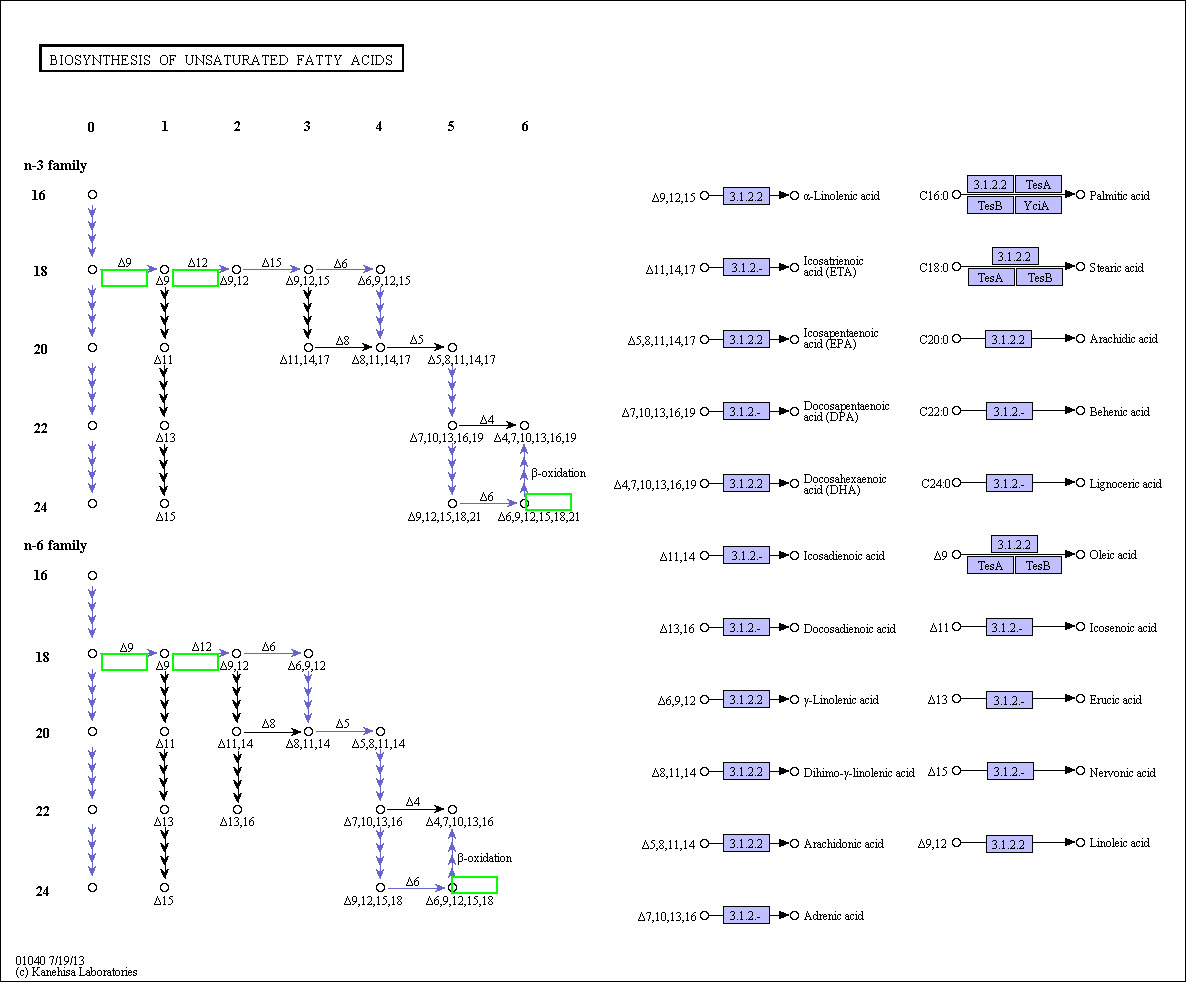

Supplement: Supplementary file 2 — Figure S2. Down-regulated DEGs involved in the biosynthesis of unsaturated fatty acids pathway. (JPG 220 kb) [file 12870_2019_1797_MOESM2_ESM.jpg]

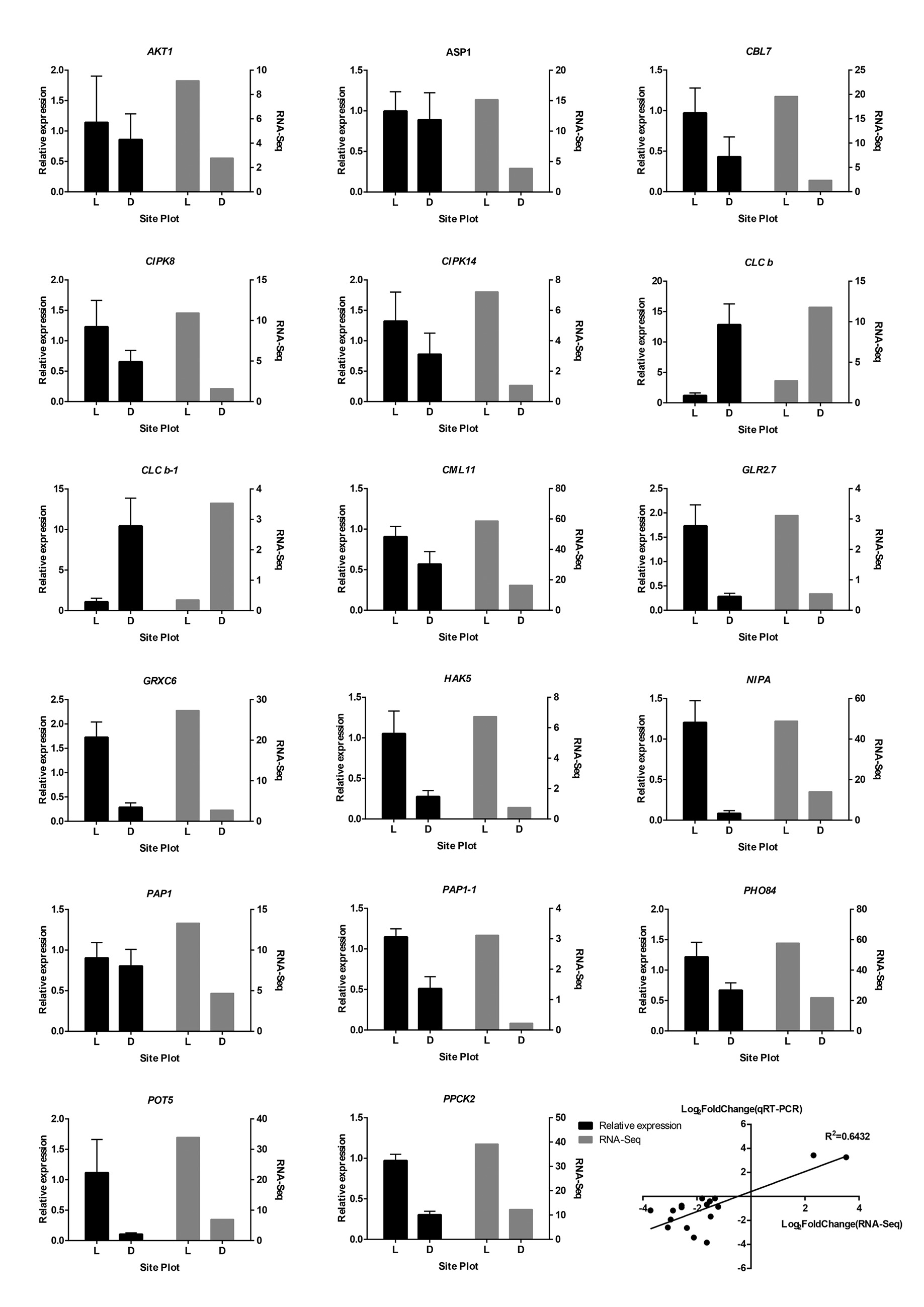

Supplement: Supplementary file 3 — Figure S3. The validation of DEGs listed in Table 4. The figure combined bar graph of gene expression and the scatter diagram of correlation analysis between RNA-seq and qPCR. DEGs involved were mentioned in Table 4. (JPG 469 kb) [file 12870_2019_1797_MOESM3_ESM.jpg]

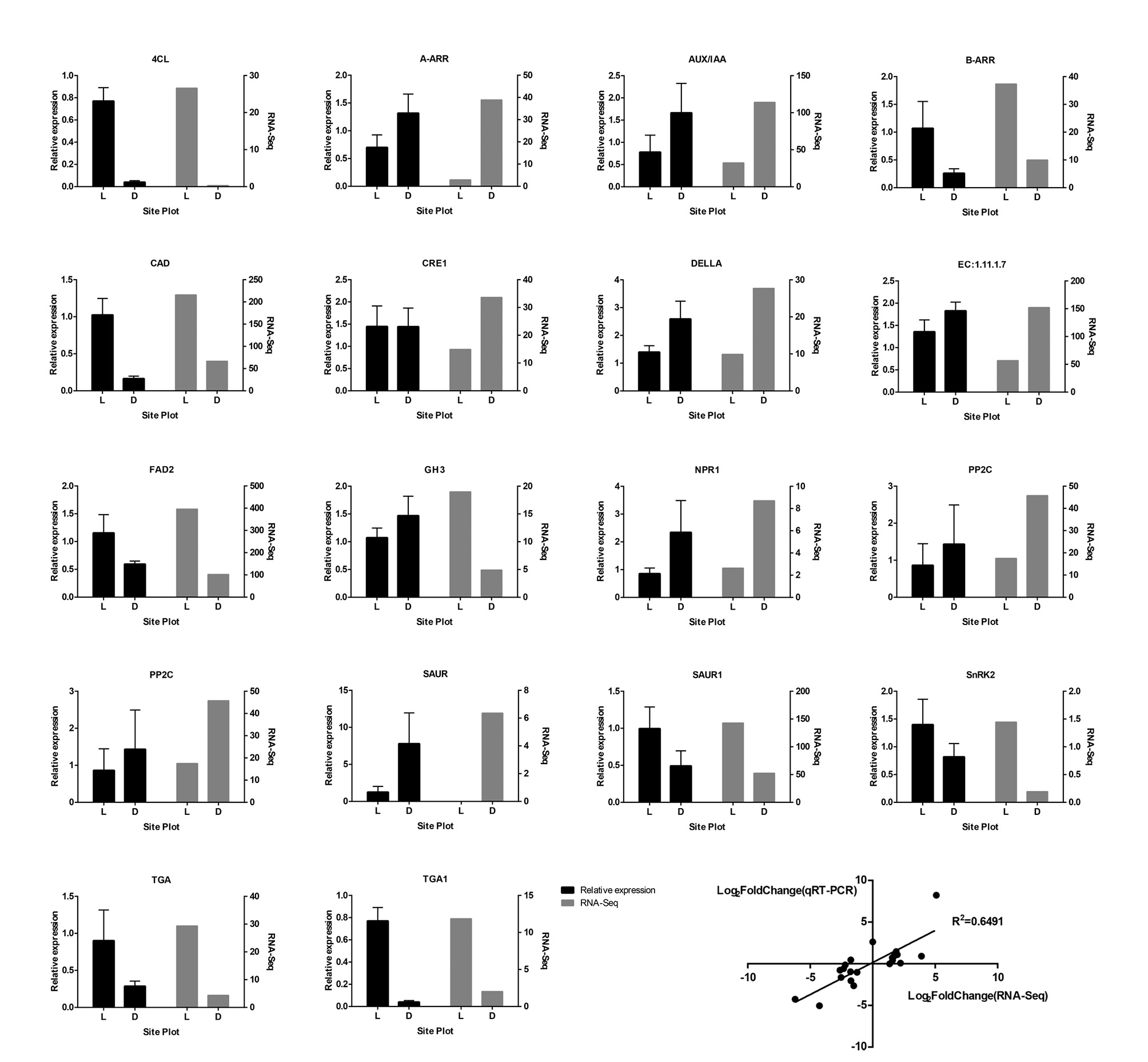

Supplement: Supplementary file 4 — Figure S4. The validation of DEGs in KEGG pathways. The figure combined bar graph of gene expression and the scatter diagram of correlation analysis between RNA-seq and qPCR. DEGs involved were enriched in the pathways of biosynthesis of unsaturated fatty acids, phenylpropanoid biosynthesis and plant hormone signal transduction, respectively. (JPG 392 kb) [file 12870_2019_1797_MOESM4_ESM.jpg]
